# Risks associated with melamine and related triazine contamination of food

**DOI:** 10.3134/ehtj.10.005

**Published:** 2009-11-10

**Authors:** RE Baynes, JE Riviere

**Affiliations:** Center for Chemical Toxicology Research and Pharmacokinetics, College of Veterinary Medicine, North Carolina State University, Raleigh, NC, USA

## Abstract

Recent adulteration of milk products with melamine (ME) in several countries caused adverse health effects and even deaths in infants. Earlier, in 2007, contamination of pet food with ME and its related contaminants was associated with many clinical cases of canine and feline nephrotoxicity, and in some cases mortality. ME is a triazine compound that is often detected with other triazine analogs such as cyanuric acid. As is the custom in some livestock operations, the contaminated pet food was mixed with feed intended for the swine and poultry industry. This practice has raised several questions as to whether ME and its related triazines would adversely affect the health of these food animals, and whether meat products derived from swine and poultry could contain high-enough levels of these contaminants to warrant public health concern. Data for this review article were obtained from recent research efforts in our laboratory, peer-reviewed publications cited in PubMed, and information available at USDA, US FDA, and WHO websites. The primary issues discussed are related to (1) the chemistry and interactions between ME and its triazine analogs; (2) reported animal and human exposures with possible pathways through which ME can enter the human food chain; (3) mammalian toxicology; (4) comparative pharmacokinetics (PK) and modeling strategies used to predict residue levels; and (5) emerging issues and management strategies.

## Chemistry of melamine (ME) and related analogs

**Figure 1:**
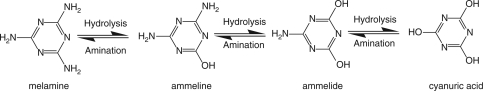
Chemical structure of melamine and related contaminants, and chemical processes that lead to conversion of melamine to different triazine analogs.

**Figure 2:**
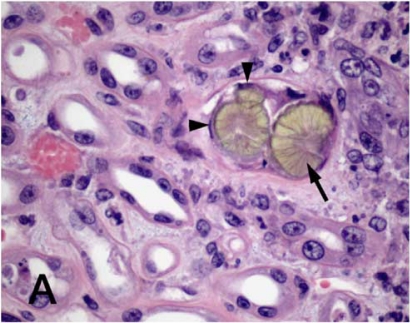
Light micrograph illustrating characteristic melamine-containing crystals (arrow) occluding the lumen of a renal tubule and necrosis of tubular epithelial cells (arrowheads).

ME (2,4,6-triamino-s-triazine) is a chemical intermediate used to manufacture amino resins and plastics in the United States of America and elsewhere. The pK_b_ (dissociation constant for a weak base) of ME is nine, because of the several amino groups that confer basic properties. ME is used in making plastics and in other industrial processes, while the related triazine compound sometimes detected with ME, CA, is used in pool chlorination. CA, as well as other chemically related triazines (Table 1), were also by-products of plastics manufacturing. CA was also detected in feed and food products during recent ME exposure incidents. ME and its related triazines have been evaluated as an alternative form of nitrogen fertilizer for plant growth. Figure 1 depicts the chemical structure of these triazines and demonstrates how they can change from one form into another via hydrolysis and/or amination. This can result in a mixture of ME and its related triazines, which can cause renal toxicity, which will be described in more detail later in this paper. These chemical reactions have been well characterized in test tube experiments and they can also occur as a result of incomplete reactions during industrial production of ME. Evidence of bacterial degradation ^1, 2^ of ME and other *s*-triazine herbicides suggests that several of these reactions can occur in the gastrointestinal tract and/or other biological fluids. Mixtures of ME and CA can result in ME cyanurate crystal formation *in vitro*, in test tubes and in the kidney (Figure 2), the target organ for the ME-associated outbreaks of renal disease in pets and human infants in recent years.^3, 4^ Prior to these events, chemists had extensively reported on the complementary hydrogen bonding between ME and CA in these crystals,^5^ and that formation of such insoluble complex crystals is pH-dependent.^6^

**Table 1:** Physicochemical properties of melamine and its analogs

*Properties*	*Melamine*	*Cyanuric acid*	*Ammelide*	*Ammeline*
Molecular Weight	126.121	129.075	128.09	127.105
pK_a_	5.35	6.88–13.5	NA	9.0
pK_b_	9.0	NA	NA	NA
Solubility in water (g/l)	3.6	2.0	0.1	0.1
Log *P*	−0.17	0.61	0.35	−0.19

## Methods

Data for this review article were obtained from recent research efforts in our laboratory, peer-reviewed publications cited in PubMed (http://www.pubmed.gov), and information available from websites of the OECD (http://www.inchem.org/documents/sids/side/108781.pdf), the US FDA (http://www.foodsafety.gov/-dms/melamine.html; wwwfda.gov/OHRMS/DOCKET/98fr/07n-0208-ra00001.pdf), and the WHO (http://www.who.int/foodsafety/fs_management/Melamine_3.pdf). Literature searches produced relevant information available in the public domain up until February 2009.

The PK analyses described in this review involved classical PK and physiologically-based PK (PBPK) modeling techniques, which are described in more detail in excellent PK texts.^7–9^ PK modeling is used in the risk assessment of toxic substances to link internal dose concentrations at tissue target sites with the exposed dose. In brief, classical PK usually involves obtaining plasma or tissue concentrations of a chemical over a selected time period and plotting the data on the logarithmic *y*-axis to demonstrate a linear relationship between plasma or tissue concentrations and time. Classical PK analyses usually involve use of a software package (for example, WinNonLin from Pharsight, Mountain View, CA, USA) and apply several compartmental and non-compartmental modeling approaches. These analyses determine PK parameters such as area under the curve (AUC), steady-state volume of distribution (Vss), half-life *(T*_1/2_), mean residence time (MRT), total body clearance (Cl), renal clearance (Cl_r_), absolute bioavailability (*F*), and absorption rate (Ka). Model predictions versus observed plasma or tissue concentrations can be shown on a graph to illustrate a model's goodness of fit. Statistical estimates of goodness of fit are often reported as R^2^, *F* test, Aikake's Information Criterion and Schwarz's criterion.

PBPK models can also be predictive of a chemical's disposition, but they are based on physiological mechanisms. This modeling process is, however, more data intensive than classical PK analyses, and utilizes a series of mass-balance equations. PBPK models link together tissue and plasma compartments using parameters that are physiological (for example, blood flow and tissue volumes), physiochemical (for example, tissue:blood partition coefficients), and biochemical (protein binding, metabolism) in nature. The mathematical modeling and simulation software AcslXtreme (Aegis Technologies Group Inc., Huntsville, AL, USA) can be used for model simulations, parameter estimation, sensitivity analysis, and Monte Carlo analysis. These PBPK models can be easily designed to incorporate multiple routes of chemical administration to predict internal dose metrics for human and animal exposure, and also for interspecies extrapolation of model results in human health risk assessment.

Human health risk assessments of chemical substances usually involve gathering all the relevant toxicological data from human epidemiological studies and animal studies. In brief, the primary focus in this exercise is to define a quantitative relationship between the dose of a chemical and an adverse health effect, and then to determine the exposure level at which the human population is least likely to be at risk. Human exposure data are often not available, and animal data need to be utilized in the assessment. With adequate animal datasets, dose–response modeling can be utilized; however, the risk assessor often resorts to a default method known as the no observed adverse effect level (NOAEL) approach, put in place to deal with the problem of having limited dose–response data. As the name indicates, the chemical dose that causes no adverse effect is selected for further computation of the risk. This NOAEL is then modified by several default uncertainty factors (×10, ×100, or ×1000) to account for interspecies differences (that is, to extrapolate the data from animals to humans) and to account for intra-species differences. Based on the above brief description of PBPK modeling, this approach can be used as an effective tool to reduce much of the uncertainty associated with NOAELs, and improve the quality of risk assessment.

## Exposure in livestock and humans

During the 2008 outbreak of ME nephrotoxicity in China, infants were exposed to milk products contaminated with ME at concentrations ranging from 0.1 p.p.m. to a maximum of 2563 p.p.m. Dietary exposure was estimated to range from 8.6 to 23.4 mg/kg b.w./day, based on median ME concentrations linked to the adulteration of a specific infant formula.^3^ More than 294,000 infants in China were exposed to these contaminants, of which more than 50,000 were hospitalized with nephrolithiasis and renal failure. At least six deaths were documented in 2008. The age-specific incidence for the illness was 85% for <2-year-olds, 17% for 2–3-year-olds, and 0.8% for >3-year-olds.^10^

In Hong Kong, approximately 3170 children were exposed to low doses of ME in the range of 0.01–0.21 mg/kg b.w./day.^11^ Of the approximately 3170 children (<12 years old), one child had confirmed renal stones, seven were suspected of having renal deposits, and 208 were positive for hematuria. No renal obstruction or acute renal failure was reported in the children exposed to these low doses.

Apart from the intentional adulteration of food products in Asia, ME could potentially migrate from plastics. This is estimated to result in a baseline exposure of 0.0019–13 µg/kg b.w./day for ME and 70 µg/kg b.w./day for CA.^3^ ME will continue to be a public health concern because of its many uses in the manufacturing and agrochemical sector. In late 2008, the US FDA detected ME (0.137 p.p.m.) and CA (0.247 p.p.m.) in several samples of infant formula in the USA. Only some of the above exposure assessments reported exposure to mixtures of ME and CA, which is believed to be more important from a public health perspective than from the viewpoint of exposure to these individual chemicals. There are no published data to indicate what threshold ratio of ME-to-CA mixture can result in nephrotoxicity.

Swine feed can contain 5–10% of pet food scraps. However, US FDA investigations during the recent ME contamination incident determined that some swine herds were fed 50–100% of pet food scraps.^12^ The concentration of the triazines in these pet food scraps ranged from 9.4 to 1952 p.p.m. for ME, while the highest values for CA, ammelide, and ammeline were 2180, 10.8, and 43.3 p.p.m., respectively. The final swine feed samples ranged from 30 to 120 p.p.m. ME, with triazine analogs also being present at lower concentrations. Assuming that the average food consumption of an average market-weight pig of 91 kg is 4% body weight, this can result in pigs being exposed to five mg/kg b.w. of ME using the high-end concentration (120 p.p.m.) of confirmed swine feed samples, or 78 mg/kg b.w. of ME using the highest concentration in *pet food scraps*. At the time of the US FDA investigation, the limit of detection (LOD) for the analytical method was 50 p.p.b. (0.05 p.p.m.) for ME only. Tissues from pigs believed to have been exposed to contaminated pet food scraps rarely contained ME levels above 50 p.p.b. There were few details regarding exact concentrations and duration of exposure, which would allow for a reliable estimate of correlation between ME exposure and tissue levels of ME.

Finally, it should be noted that this international incident of ME contamination prompted many countries to establish action limits for this contaminant and related analogs. For example, a maximum ME limit of one p.p.m. was established for infant formula and a maximum ME limit of 2.5 p.p.m. was established for milk products. Some countries have established a limit of reporting (LOR) due to setting of maximum limits as above instead of an analytical level of determination such as the LOD or limit of quantitation (LOQ). The reader should also be aware that, in most of the surveys, the LOD or LOQ varies not only across food matrices but also across laboratories and countries. The reporting of positive ME samples should therefore be accompanied with the LOD or LOQ of the analytical method used during the survey. For example, the Chinese Center for Disease Control and Prevention reported ME concentrations in infant formula from Beijing and Gansu provinces ranging from less than 0.05 to 4700 p.p.m. The presence of positive samples with levels less than 0.05 p.p.m. suggests that the LOD for the analytical method was less than 0.05 p.p.m. Health Canada reported ME concentrations ranging from 0.0043 to 0.346 p.p.m., with an LOD of 0.004 p.p.m. The US FDA has also reported positive milk formula samples at 0.137–0.206 p.p.m., although these positive values were below the LOQ of 0.25 p.p.m. The above are just examples of surveys in several countries where positive samples are often below the LOQ and in most cases below the LOR.

## Mammalian toxicology

### Animal studies

Previous experimental studies determined that toxicity to pure ME in mammals should be of minimal concern, a finding supported by the large oral lethal dose (LD50) of 3161 mg/kg in rats.^13^ Several other studies cited in this OECD report describe a dose–response relationship between ME and kidney stone formation in Fischer 344 rats given 63–1000 mg/kg ME by oral feed for 13 weeks. Similar dose–response relationships were also demonstrated in B6C3F1 mice fed ME for 13 weeks, but at a higher dose range than that tested in the Fischer 344 rats. The diuretic effects of this chemical have been reported in rats and dogs.^14^

Male Fischer 344 rats exposed to extremely high doses of ME in their diets for 36 to 103 weeks developed bladder stones as well as transitional-cell carcinoma in statistically significant proportions compared with controls.^15–17^ These studies consistently demonstrated that male rats, more so than female rats, were predisposed to developing bladder tumors. When B6C3F1 mice were exposed to higher concentrations than the rats for the same exposure period, only acute or chronic inflammation, epithelial hyperplasia, and bladder stones (but no bladder tumors) were observed. The carcinogenic effects observed in the rats were strongly correlated with the presence of urinary bladder stones, and there is no evidence of direct molecular interactions attributed solely to ME. Genotoxicity studies also demonstrated that ME is very unlikely to be a mutagen.^15, 18^ There are, however, several reports suggesting that certain fatty acids can modulate murine urinary proliferative lesions induced by ME.^19, 20^

There is sufficient evidence to suggest that the mechanism of renal toxicity in dogs and cats is associated with crystallization of triazines in the kidney, followed by renal failure, as observed with the adverse effects recently reported in pets in the USA.^4, 12, 21, 22^ In all diagnostic reports about these cases, ME alone does not appear to be toxic, but exposure to ME and CA as a mixture, at doses ranging from 32 to 181 mg/kg per chemical for about two days, caused characteristic yellow-brown crystals in distal renal tubules and collecting ducts, leading to nephrotoxicity.^23^ These animals all had markedly high concentrations of blood urea nitrogen (BUN), creatinine, and isosthenuric urine. Often the urine contained casts, leukocytes, erythrocytes, and yellow-brown crystals. In three dogs that were exposed to pet food suspected of contamination, infrared spectroscopy and scanning electron microscopy with energy-dispersive X-ray analysis were able to distinguish between calcium oxalate crystals and ME-containing crystals.^24^ In addition, histochemical staining of kidney sections with Alizarin Red S was used to demonstrate the presence of divalent cations associated with calcium oxalate and calcium phosphate crystals, while Oil Red O stains with ME-containing crystals confirmed the presence of ME. The only domestic or companion animal study that demonstrated similar nephrotoxicity after exposure to apparently a ME-only gavage involved sheep exposed to high doses of approximately 200 mg/kg for several days.^25^ Although published some time ago, the observations from this study suggest that rumen metabolism may play a role in the conversion of ME to analogs that result in crystal formation and subsequent nephrotoxicity in the sheep.

Recent studies conducted by the US FDA^26^ have reported that no microscopic crystals were observed in the kidney when fish or swine were exposed to either ME or CA, at 400 mg/kg of either contaminant for three days with a one day withdrawal. However, when these two contaminants were fed to either species as a mixture for the same time period at a high dose of 400 mg/kg for each contaminant, crystals were observed in the kidney. Interestingly, ME–CA crystals were also detected in the intestines of the pigs that were exposed to a 1:1 ratio of ME+CA. Furthermore, tissue levels of ME or CA were greater when given as individual chemicals, but these levels were significantly lower when the chemicals were given as a mixture, suggesting that crystallization in the intestines may have limited bioavailability. However, bioavailability was not assessed, and neither were urine or blood levels of either triazine. Dose composition could not be predicted from crystal composition, which had a consistently higher ME/CA ratio than the equimolar (1:1) dose used in this study.

Several biomarkers of renal toxicity (BUN, creatinine, creatinine clearance) are responsive to exposure to ME–CA mixtures.^27^ This study^27^ demonstrated significant changes in BUN, serum creatinine, and creatinine clearance when rats were exposed to single-dose mixtures (1:1), or three doses of mixtures (1:1 or 10:1); it also demonstrated a potential dose–response relationship across dose and ME/CA ratios. This study was also unable to demonstrate the *in vitro* cytotoxicity of individual triazines in canine kidney cells (MDCK) and feline kidney cells. Furthermore, the study did not evaluate the cytotoxicity of ME–CA mixtures in these *in vitro* systems.

### Human studies

Prior to the ME-poisoning cases seen in China in 2008, there was no evidence demonstrating the adverse effects of exposure to a mixture of both contaminants in humans—even though crystal obstructive nephropathies leading to acute renal failure are well documented in humans following exposure to several drugs such as some sulfonamides (for example, sulfadiazine), acyclovir, or methotrexate.^28, 29^ The mechanisms of crystal-induced inflammation in diverse cell types caused by various crystals such as calcium oxalate, calcium phosphate, and urate have also been well characterized.^30^ However, the mechanisms associated with the adverse effects of ME cyanurate crystals on renal tubular cells have not been investigated. Cases of possibly similar pathogenesis, where nephroliths form to cause subsequent renal toxicity, have been associated with human exposure to triamterene and xemilofiban/orbofiban These are chemicals with nitrogen-ring-based structures whose toxicity is highly variable and is dependent on concentration as well as urine variables that promote precipitation in the tubular fluid.^31^ Because of the similarity of clinical syndromes between these drugs and ME, one could hypothesize the plausibility of a similar mechanism. More importantly, other nephrotoxins that do not share the same chemical structure as ME or drugs with nitrogen-ring base structures result in different signs of clinical toxicology.

Several recent publications have attempted to correlate the known exposure levels of ME in powdered milk,^32^ and ME levels in urine,^33^ with urolithiasis in infants in China. The study by Guan *et al*.^32^ demonstrated that infants exposed to the high-ME formula (>500 p.p.m. ME) were seven times as likely to have stones as those exposed to ME-free formula, and preterm infants were 4.5 times as likely to have stones as full-term infants. Urinalysis did not appear to be an adequate method of screening for ME-associated urinary stones. Biomarkers of early renal disease suggest that ME-related stone formation appeared to be associated more with glomerular dysfunction rather than tubular dysfunction. The study by Lam *et al*.^33^ determined that urine ME levels could be a biomarker of residual ME load in the body, and found a strong correlation between renal stone size and urinary ME levels. However, there was no such correlation for CA levels in urine.

## Pharmacokinetics

The most complete PK modeling study was performed in male Fischer 344 rats exposed to a single oral dose of 0.38mg (1.3 mg/kg b.w.) of radiolabeled ^14^C) ME.^34^ In a one-week feeding study in male Wistar rats, ME was not absorbed in the stomach, but exponential absorption occurred in the small intestine.^35^ The former study demonstrated that plasma half-life was about three hours, more than 90% of the oral dose was eliminated in the urine within 24 h, and more ME was recovered in the kidney and bladder than in other tissues. These PK findings provide some support for the toxicity reported in rats, which is thought to have led to the adverse effects observed in pets exposed to ME-contaminated pet food and infants exposed to contaminated milk.

**Figure 3:**
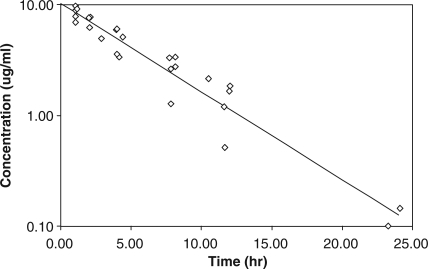
Plasma concentration–time profiles of melamine in swine. The solid line indicates plasma concentrations generated from the one-compartment model and observed data points. *Source*: Baynes *et al*.^36^

Our laboratory was the first to describe the PK of ME given as a single intravenous (IV) dose to pigs in order to obtain basic PK parameters.^36^ A one-compartment, first-order kinetic, IV bolus model was found to be the best model for the data (Figure 3), and first-order elimination was assumed based on previously reported rodent data.^34^ The estimated half-life, clearance, volume of distribution at steady state, and AUCs are listed in Table 2. Individual parameter values were calculated from each pig. The volume of distribution of ME in pigs (0.61+0.04 l/kg) was close to that of total body water, suggesting that the distribution of this chemical for the most part may be limited to the extracellular fluid compartment. The limited distribution in the pig is consistent with data from the earlier rat study,^34^ which demonstrated that tissue distribution may be limited to the total body water, with ME possibly binding to the kidney, which is the organ primarily responsible for its clearance. This is a safe assumption for the pig, as ME is not metabolized by the liver in other animals.^37^

**Table 2:** Pharmacokinetic parameters for melamine in pigs following IV dose of 6 mg/kg melamine

	T_*1/2*_*(h)*	*Cl (l/h/kg)*	V_*ss*_*(l/kg)*	*AUC (h mg/ml)*	*Kel (1/h)*	*MRT (h)*
Mean	4.07	0.11	0.61	59.26	0.18	5.87

Abbreviations: AUC, area under the curve extrapolated to infinity; Cl, clearance; Kel, elimination rate constant; MRT, mean residence time; *T*_1/2_, half-life; Vss, volume of distribution.

ME appears to be cleared rapidly (*T*_1/2_=4.07±0.39 h) and monoexponentially in the pig, with a half-life that is approximately 1.5 times longer than that seen in the rat (*T*_1/2_=2.71 h). This is, in turn, primarily due to an almost five-fold greater renal clearance in the rat than in the pig, in spite of the apparent volume of distribution in the rat study (1.8 l/kg) being three times greater than that in the pig (0.61 l/kg). Clearance mechanisms for ME and its analogs have not been characterized in various animal species; however, based on its physicochemical properties as a small polar molecule, and its clearance values (0.11 l/h/kg or 27 ml/min), ME can be assumed to be primarily cleared by renal filtration in the pig. The fact that the glomerular filtration rate (GFR) of our weanling pigs (15 kg) is approximately 31–42 ml/min^38^ supports this preliminary assessment.

Pigs' blood would be cleared of about 99% of the ME dose within seven half-lives or 28 h after exposure, and this blood concentration should be below the safe level of 50 ng/ml (50 p.p.b.) proposed by the United States Food Safety Inspection Service (US FSIS)^39^ for a dose of six mg/kg. This proposed safe level is based on the LOQ for the assays developed by US FSIS. In the likely scenario that pigs were accidentally exposed to a large oral dose that resulted in a maximum plasma concentration of 10 µg/ml, as in our IV study,^36^ based on extrapolation of our PK model, it would take an estimated 28.6 h for blood ME levels to deplete to the proposed safe concentration. The earlier rat studies^34^ provided an incomplete dataset to estimate depletion of the chemical from the body, because the selection of inadequate sample times did not allow for appropriate estimation of the ME half-life in the rat kidney. Furthermore, all these pig and rat studies used exposures to pure ME; so the effects of concurrent triazine exposure (ME+CA) on ME disposition and tissue withdrawal time are not known.

Although not thoroughly investigated, the available evidence suggests that ME does not undergo significant hepatic metabolism and should, for the most part, be readily cleared solely by the kidney. However, biliary excretion cannot be ruled out and requires further investigation. ME can be broken down by hydrolytic enzymes and bacteria to CA, ammelide, and ammeline.^40^ There is reason to believe that digestive enzymes can have a similar effect on ME, with the resulting metabolites appearing in the urine and feces.

At low doses of five mg/kg, CA appears to be readily absorbed via the gastrointestinal tract of rats and largely eliminated in the kidney without being metabolized, resulting in a half life of 0.5–1.0 h.^41^ But at a higher dose of 500 mg/kg, the chemical is incompletely absorbed and eliminated mostly in the feces, with a half-life of 2.5 h. CA does not appear to bioaccumulate in rats given the lower dose for 14 days. Similar findings were observed in dogs given the same doses, except that elimination half-lives were estimated at 1.5–2.0 h with no metabolism.

As clearance of ME and its analogs is dependent on elimination via the kidney, there is the possibility that ME cyanurate crystal formation and related renal pathologies in the renal tubules could reduce the renal clearance of ME and its analogs. Numerous clinical PK studies have demonstrated the influence of compromised renal clearance on plasma and tissue concentrations of various drug classes following renal disease.^7, 8^ Ethylene glycol-induced nephrotoxicity is well documented in the literature, and more recent work described increased sensitivity to oxalate crystal formation in some rats, leading to degeneration of the renal tubule epithelium.^42, 43^ Based on the data from this study, it appears possible that ME cyanurate crystal formation in the renal tubule can reduce the kidney's capacity to excrete these triazine contaminants (oxalic acid or ME/CA), and this further exacerbates toxicity. However, a more recent study42 demonstrated no statistically significant differences in renal clearance between treated animals and controls, and renal clearance of oxalic acid appears to be unaffected in humans with urolithiasis, glomerulonephritis, or polycystic kidney disease.^44^ ME cyanurate crystals in swine or humans may be different: unlike normal crystals in dogs, which may cause chronic obstruction, ME cyanurate crystals were acutely lethal and caused acute renal failure. As a further note on how oxalic acid is cleared by the kidney across different species, ^14^C-oxalate-to-inulin renal clearance ratios in healthy rats were lower than one, indicating that a fraction of the filtered oxalate was reabsorbed,^42^ contrary to what happens in humans, sheep, and dogs.^45, 46^

**Figure 4:**
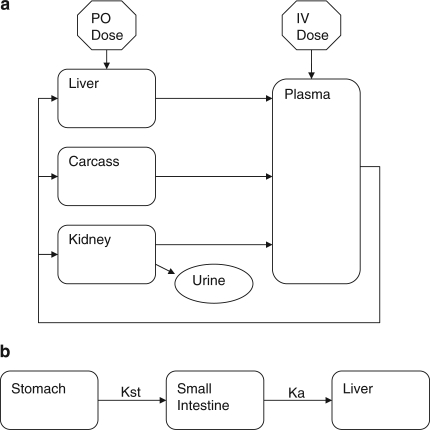
**(a)** Schematic illustration of the physiologically based pharmacokinetic model of melamine. This model was used in both rat and porcine species. The arrows represent mass transfer of melamine via blood flow. **(b)** Schematic representation of the chronic oral dosing regimen. Kst and Ka represent the rate of gastric emptying and rate of absorption, respectively. *Source*: Buur *et al*.^47^

**Figure 5:**
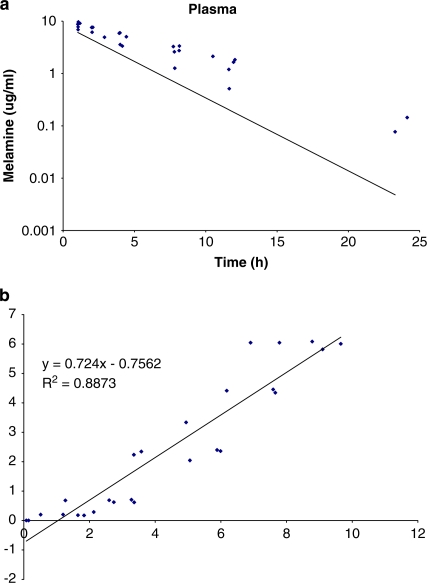
Validation data for porcine melamine PBPK model against porcine plasma data. (**a**) Model simulation (solid line) and observed data (squares) and (**b**) regression analysis between model simulation and observed data. *Source*: Buur *et al*.^47^

Our laboratory has also modeled ME disposition in rats following an oral bolus exposure.^47^ This four-tissue compartment PBPK model (Figure 4) compared somewhat favorably, based on *R* ^2^ values (*R* ^2^=0.59–0.76), with limited tissue data from the rat study.^34^ Similar strategies were used to model ME disposition in pigs following IV bolus exposure with better predictions (*R* ^2^=0.89) for plasma data (Figure 5). No tissue samples were analyzed in this preliminary IV study in pigs. However, the PBPK model was used to predict a tissue withdrawal time of 21 h for a 5.12 mg/kg single oral bolus, although we have no *in vivo* pig studies to validate these estimates. Further studies are also needed to test our model assumptions on parameters such as species differences in tissue:plasma partitioning, rates of intestinal absorption, and renal clearance mechanisms such as differences in GFR, dose linearity, and tissue dosimetry.

The inherent value of a PBPK modeling method is that it incorporates species-specific physiological and biochemical processes that can be evaluated to determine their influence on tissue disposition of a chemical. This approach is not driven by tissue time–concentration curves, which is typical of the classical compartmental PK or data-based PK modeling, which assume a specific model structure and derive model parameters by curve fitting. This classical approach assumes that absorption, distribution, metabolism, and excretion processes are first order, but does not always take into account potential saturation clearance limitations for some chemicals, or that blood flow can limit clearance.

In PBPK modeling, once a conceptual and realistic model has been defined, physiological and biochemical data parameters such as blood flow, tissue volumes, metabolic constants, and blood:tissue partitioning values can be obtained from the literature and/or from separate *in vitro* laboratory experiments. Once the model has been developed, one can determine the influence of variability and uncertainty of physiological parameters on the model output. For example, swine body weight and blood flow vary within a population; therefore, a method known as Monte Carlo technique can be used to understand the variability of the response or kinetic behavior of these triazine compounds.

## Human health risk assessment

The risk assessment of ME is complicated by the fact that differential toxicity has been reported across various animal species. It is reasonable to assume that species-specific toxicokinetics and renal toxicology may contribute to reported species differences. Many human and animal studies do not report the purity of the ME exposure and/or the purity of the urine samples to ascertain whether CA or other triazines were present in these matrices. Current human health risk assessments are based on a rat study, which has derived a NOAEL of 63 mg/kg b.w./day based on stone formation in rats.^10^ An uncertainty factor of 100 is used for inter-species and intra-species extrapolations, and an additional safety factor of 10 for combined exposures to ME and CA. We note that it is mixtures of ME and CA that have been implicated in the recent human and pet nephrotoxicity incidents. From these calculations, the total daily intake was determined to be 0.063 mg/kg b.w./day, or a daily intake of 3.78 mg/person/day. A safe level or tolerance level in food was calculated at 2.5 p.p.m., assuming a food consumption rate of 1.5 kg/day. This calculation was also modified for infants weighing three kg and consuming 0.15 kg of food; that is, the new safety level for ME would be 1.26 or 1.00 p.p.m., to provide an additional margin of safety.^10^ In other words, if 100% of an infant's diet was contaminated at a level of 1.26 p.p.m. of ME (0.189mg ME per 0.15 kg food), the daily intake would equal 0.063 mg/kg b.w./day, suggesting that the 1.00 p.p.m. safety level provides an additional margin of safety. These are currently set as interim safety levels and will probably be revised as we gain a better understanding of the toxicokinetics and toxicology of these chemical mixtures in sensitive subpopulations.

## Emerging issues and management of contamination

There are now significant human epidemiological^32, 33^ and animal experimental data^26, 27^ that humans or animals exposed to mixtures of ME and CA in their diets are very likely to develop crystal nephropathy that can result in acute renal failure and possibly death. The human studies did not report whether infants were exposed to mixtures of ME and CA. However, the animal experimental studies strongly suggest that the affected infants were most likely exposed to mixtures of both triazines in the milk formula. It is very likely that adulterants such as ME and related triazines will be used as a cheap nitrogen source in the face of limited protein/feed supply. Future protein substitutes in human food and milk may contain these triazines either individually or as a mixture. Furthermore, the widespread use of related triazines in numerous applications (for example, as herbicides) suggests the potential for significant occupational exposures and environmental exposure, perhaps through contamination of water sources. This highlights the need to better understand the disposition of and toxicological interactions between these triazines in animals as well as humans.

Management of any future outbreaks linked to ME contamination of animal feed and human food will require access to state-of-the-art screening and confirmatory analytical methods to identify and quantify the presence of ME and its related triazines, both in food items and in biological matrices. The literature^48–50^ describes numerous ELISA, LC, and GC techniques that have been successfully used to analyze these triazines in feed, blood, urine, and tissues. X-ray diffraction and Fourier transform IR spectra of crystals formed in these matrices, together with mass spectrometry, have been used and can be used for identifying crystals and confirming their chemical composition in pets and human patients. Although testing for ME is one aspect of outbreak management, identification of reliable biomarkers of exposure and toxicity could provide improved diagnostic tools during the early stages of an outbreak. To date, many of the available clinical pathology parameters of urolithiasis appear to be of limited diagnostic value in screening patients for ME-induced urolithiasis, as demonstrated in the recent study by Guan *et al*.^32^

An understanding of how these triazines are cleared from circulation in livestock can be used in managing future outbreaks and preventing adulterated food of animal origin from entering the human diet. In addition to improved chemical analytical methods for animal feed and human food analysis, PBPK modeling can be used in conjunction with residue data from tested livestock to estimate when meat and milk products are safe for human consumption. There are, however, significant gaps in data with regard to (1) PK and tissue disposition following oral exposure to ME–CA mixtures, (2) the physiological and physicochemical factors that can influence the PK of simultaneous exposure to these mixtures, (3) the ratios and doses of ME–CA mixtures that can cause nephrotoxicity, and (4) the dose– response relationship between exposure to the mixture and renal adverse effects.

Current risk assessments are based on exposures to ME alone, and not to any mixture of ME and its related triazines. There has been an increased awareness of the importance of chemical mixtures in risk assessment and most of these studies have demonstrated that single chemical toxicity data are not predictive of the toxicity seen after exposure to the same chemicals in complex mixtures. There are numerous possible interactions between the chemicals based on alterations in systemic disposition, biotransformation, or toxicodynamic interactions at the membrane, cellular, or receptor level. These interactions may be additive, synergistic, or antagonistic. Based on preliminary data presented in this review, the interaction between ME and CA may not be deemed synergistic in the classical toxicology sense, although exposure to any one of these triazines is less likely to cause adverse renal effects compared with a mixture of both triazines. There is clearly a need to increase the capacity to assess the risk to animal and human health following exposure to chemical mixtures. Exposure to a mixture of known interacting chemicals such as these triazines needs to be handled differently from that which involves single chemicals.

## Conclusions

Recent ME contamination incidents crossed international boundaries and involved contamination of not only milk products but also other food items. This review highlights human and livestock exposure to this contaminant and/or its analogs, and the adverse health effects seen in exposed infants. Performing an adequate risk assessment of this contaminant and its analogs is limited by insufficient human and animal dose–response toxicology data, among many data gaps. Although ME is not metabolized by the liver and does not appear to accumulate in the body, as it is rapidly eliminated by the kidney, there is little or no mechanistic information to guide risk assessment when a mixture of ME and CA or other analogs are simultaneously cleared by the kidney. Further work is needed to assess the carry-over ratios of ME and related analogs from feed, animal products, oral bioavailability, and renal clearance in order to better determine tissue dosimetry in the human body if a similar food contamination incident was to threaten human health again.
